# Complete Response in Metastatic Hepatocellular Carcinoma with Cardiac and Lung Involvement via Multimodality Treatment

**DOI:** 10.3390/medicina57080849

**Published:** 2021-08-20

**Authors:** Kun-Feng Tsai, Jerry C. H. Tsai, Ming-Feng Li, Jimmy W. H. Tan, Chu-Kuang Chou, Huei-Lung Liang, Shan-Ho Chan

**Affiliations:** 1 Gastroenterology and Hepatology Section, Department of Internal Medicine, An Nan Hospital, China Medical University, Tainan 709, Taiwan; tsai.kf@gmail.com; 2 Department of Medical Sciences Industry, Chang Jung Christian University, Tainan 711, Taiwan; 3 Department of Radiology, New Taipei City Hospital-Sanchong Branch, New Taipei City 241, Taiwan; cryoarcher@gmail.com; 4 Department of Radiology, Kaohsiung Veterans General Hospital, Kaohsiung 813, Taiwan; hlliang@vghks.gov.tw; 5 Department of Medical Imaging and Radiology, Shu-Zen Junior College of Medicine and Management, Kaohsiung 821, Taiwan; shchan@ms.szmc.edu.tw; 6 Division of Cardiovascular Surgery, An Nan Hospital, China Medical University, Tainan 709, Taiwan; neugine@gmai.com; 7 Division of Gastroenterology and Hepatology and Clinical Trial Center, Ditmanson Medical Foundation Chia-Yi Christian Hospital, Chia-Yi 600, Taiwan; vacinu@gmail.com

**Keywords:** metastatic hepatocellular carcinoma, tumor thrombi in inferior vena cava or right atrium, multimodality treatment

## Abstract

*Background*: Until recently, advanced HCC patients with major vessel and cardiac involvement have had an extremely poor prognosis without satisfactory treatment. *Case presentation*: A 63-year-old Taiwanese male presented with metastatic HCC with RA and IVC thrombi, as well as pulmonary metastases that were successfully treated by multimodal management, encompassed by surgical thrombectomy, concurrent systemic sorafenib and locoregional therapies, and immunotherapy. The patient has achieved a complete response over the past 33 months. *Conclusions*: Through this case report, which shows a successful outcome via multimodal management, a more aggressive approach should be considered when a patient is expected to tolerate the risks and side effects of various treatments.

## 1. Introduction

Advanced hepatocellular carcinoma (HCC) patients with tumor thrombi in the inferior vena cava (IVC) or right atrium (RA) generally have an unfavorable prognosis, and there is no established management protocol for treatment [1]. In light of the poor outcomes of this condition, we utilized a multimodal approach to achieve a complete response in a patient with pulmonary metastases and tumor thrombi in IVC and RA.

## 2. Case Presentation

**Figure 1:**
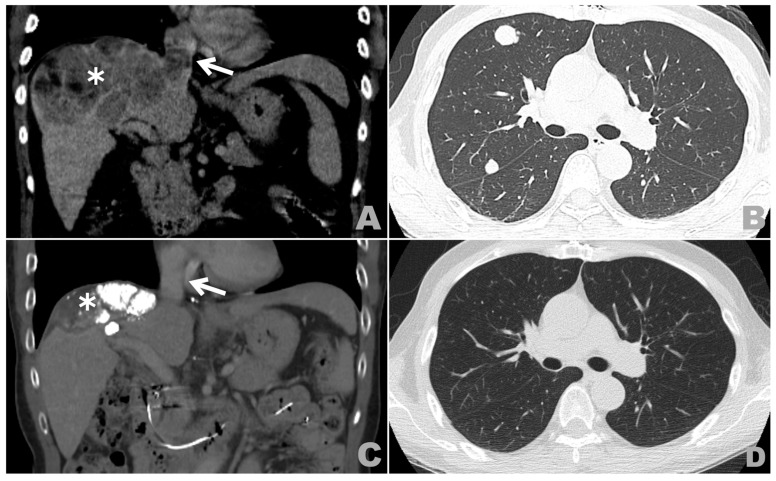
Key imaging studies with different treatment modalities: (**A**) contrast-enhanced CT of upper abdomen with coronal reconstruction at initial diagnosis shows a huge liver tumor (white asterisk, *) with tumor thrombi (white arrow) in inferior vena cava (IVC) and right atrium (RA). (**B**) Chest CT after hepatic artery infusion chemotherapy (HAIC) and drug-eluting bead transarterial chemoembolization (DEB-TACE) shows progressive metastatic lung nodules. (**C**) Follow-up contrast-enhanced CT of upper abdomen with coronal reconstruction after multimodal treatment shows tumor shrinkage with cystic necrosis and lipiodol retention (white asterisk, *) and resolution of previous RA and IVC tumor thrombi (white arrow). (**D**) Follow-up chest CT after 8 cycles of nivolumab shows clear bilateral lung fields without metastatic lung nodules.

A 63-year-old Taiwanese male presented with progressive bilateral lower limb edema, abdomen fullness, and dyspnea on exertion for 3 weeks. He had a medical history of hepatitis B virus-related cirrhosis with Child–Pugh class A liver function. An initial blood test revealed a significantly elevated hepatitis B viral load up to 45,400 IU/mL and a serum alpha-fetoprotein (AFP) level up to 6768.5 ng/mL, despite normal serum albumin, serum total bilirubin, and prothrombin time. Abdomen computed tomography (CT) disclosed a huge right lobe liver tumor up to 13 cm that was consistent with HCC enhancement patterns. Direct tumor invasions into the right portal vein, plus the middle and right hepatic veins, were detected, along with tumor thrombi within IVC and RA (Figure 1A). Furthermore, a small metastatic lung nodule measuring about 1 cm at the right lower lobe was also identified.

Anti-viral medication with Tenofovir was prescribed after the detection of a high hepatitis B viral load, and prompt surgical removal of IVC and RA thrombi was indicated after multidisciplinary discussion regarding the aggravating IVC obstruction-associated symptoms. The patient therefore underwent a cardiopulmonary bypass and median sternotomy, and RA was incised, with all IVC and RA thrombi excised en bloc under direct vision. Gross pathology examination revealed a cauliflower-like tumor, which was microscopically confirmed as poorly differentiated HCC. The patient’s symptoms were alleviated soon after surgery. As for treatment of the hepatic tumor, initial systemic treatment with sorafenib (400 mg twice daily) was given orally while localized treatment with hepatic artery infusion chemotherapy (HAIC) was also arranged concomitantly. The HAIC was a combination regimen consisting of cisplatin, mitomycin-C, and 5-fluorouracil, followed by lipiodol infusion before infusion catheter removal [2]. Mild skin eruption at his scalp was noted after sorafenib usage, which was treated with topical glucocorticoids and oral antihistamines. The skin eruption healed within 10 days.

**Figure 2:**
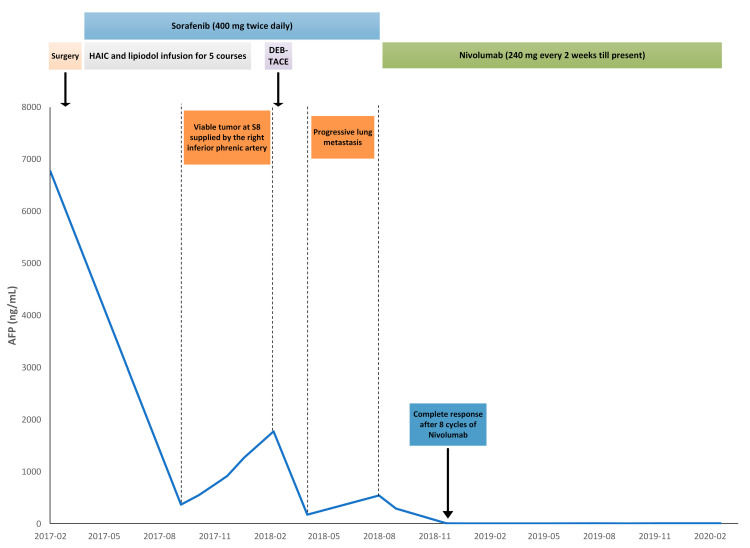
A graph demonstrating the corresponding changes in serum alpha-fetoprotein (AFP) level upon different treatment modalities prescribed throughout the clinical course.

The patient received five courses of HAIC over a period of 9 months, with a steady decline in serum AFP (Figure 2). However, a progressive viable HCC from extrahepatic blood supply was noticed on follow-up CT, and drug-eluting bead transarterial chemoembolization (DEB-TACE) loaded with doxorubicin was performed via the right inferior phrenic artery. Despite remarkable liver tumor necrosis, declined AFP, and no residual tumor thrombi after the aforementioned treatment, progressive pulmonary metastases were later discovered (Figure 1B), and sorafenib was thus switched to nivolumab (160 mg every 2 weeks, according to the dosage guideline of 3 mg/kg every 2 weeks) in order to control further pulmonary metastases. No adverse events related to nivolumab usage were observed. After eight cycles of nivolumab therapy, a complete response was finally achieved, with no viable HCC, pulmonary metastases, or tumor thrombi visible (Figure 1C,D); serum AFP level also returned to normal at 3 ng/mL (Figure 2). The patient continued with nivolumab therapy and has survived for 53 months since the initial diagnosis of advanced HCC; he has remained disease free for the past 33 months, until July 2021.

## 3. Discussion

Advanced HCCs with invasions into the IVC or right atrium are quite uncommon, accounting for only 3 to 4% of HCC patients [3]. Since macroscopic vascular invasion is a strongly negative prognostic factor for advanced HCC patients [4], the presence of IVC or RA thrombi usually translates to extremely poor prognosis, as the risks of sudden death from pulmonary embolism or acute heart failure and systemic metastasis are greatly increased. Currently, surgical removal under cardiopulmonary bypass for HCC patients with tumor thrombi in RA or IVC has been reported as a safe and effective method to alleviate the symptoms associated with venous obstruction, and it can improve prognoses. Although the median survival time can last up to 19–30.8 months in reports, how to deal with early tumor recurrence and metastatic tumors remains an unresolved issue [1,5]. Given the remarkable advances in molecular-targeted therapy and immune checkpoint inhibitors in recent years, these therapies have emerged as new cancer treatment methods against metastatic HCC [6]. Even though there are currently no established guidelines for the treatment of advanced HCC patients with IVC or RA thrombi, plus extrahepatic metastasis, a combination of treatment modalities, including surgery, locoregional therapies, radiotherapy, and target or immunotherapy, turned out to be a promising solution for our patient.

In this patient, the surgical removal of IVC and RA thrombi was conducted first to relieve the symptoms caused by IVC obstruction, as well as to mitigate the risk of sudden death from tricuspid valve occlusion or pulmonary embolism. Considering the patient had a Child–Pugh A liver reserve, subsequent systemic treatment of sorafenib plus locoregional therapy with HAIC, followed by lipiodol infusion and DEB-TACE, were applied to induce extensive hepatic tumor necrosis and shrinkage [7,8]. Lastly, nivolumab was used as the sole immunotherapy because it was the only approved agent for metastatic HCC at that time (2018) in Taiwan; it was administered and maintained as a successful treatment for the patient’s sorafenib-resistant pulmonary metastasis [9].

## 4. Conclusions

In summary, we report an advanced HCC patient with tumor thrombi in RA and IVC, plus pulmonary metastases, who attained a complete response after receiving multimodal treatment. Although current treatment algorithms favor palliative treatment for late-stage HCC, a more aggressive approach with multimodal management should be considered as an alternative option when a patient is capable of tolerating the risks and side effects of the various aforementioned treatments; these treatments can reasonably improve survival over any other single therapy in patients with advanced HCC.

## Data Availability

The data presented in this study are available on request from the corresponding author.
